# Interferon-γ enhances the antifibrotic effects of pirfenidone by attenuating IPF lung fibroblast activation and differentiation

**DOI:** 10.1186/s12931-019-1171-2

**Published:** 2019-09-11

**Authors:** Tuong N. Vu, Xuesong Chen, Hussein D. Foda, Gerald C. Smaldone, Nadia A. Hasaneen

**Affiliations:** 1 Pulmonary, Critical Care and Sleep Division, Department of Medicine, Stony Brook Medicine, Health Science Center, State University of New York at Stony Brook, HSC T17 Room 040, Stony Brook, NY 11794-8172 USA; 2 Department of Medicine and Research, VAMC Northport, Stony Brook, NY USA

**Keywords:** Fibroblast activation, IPF, IFN-γ, Pirfenidone, MMPs, TIMPs

## Abstract

**Background:**

Idiopathic pulmonary fibrosis (IPF) pathogenesis involves multiple pathways, and combined antifibrotic therapy is needed for future IPF therapy. Inhaled interferon-γ (IFN-γ) was recently shown to be safe and without systemic effects in patients with IPF.

**Aim:**

To examine the in vitro effects of individual and combined treatment with IFN-γ and pirfenidone (PFD) on normal and IPF fibroblast activation and extracellular matrix remodeling after TGF-β1 and PDGF-BB stimulation.

**Methods:**

IPF and normal human lung fibroblasts (NHLF) were treated with IFN-γ, PFD or a combination of both drugs in the presence of either TGF-β1 or PDGF-BB. The effects of TGF-β1 and PDGF-BB treatment on cell viability, proliferation, differentiation and migration were examined. The expression of collagen 1, matrix metalloproteinases (MMPs) and tissue inhibitors of MMP (TIMPs) was analyzed using qPCR, Western blotting and gelatin zymography. Total collagen content in conditioned media was also measured using a Sircol assay.

**Results:**

Compared to that of PFD, the effect of IFN-γ in downregulating normal and IPF lung fibroblast differentiation to myofibroblasts in response to TGF-β1 was more potent. Importantly, the combination of IFN-γ and PFD had a possibly synergistic/additive effect in inhibiting the TGF-β1- and PDGF-BB-induced proliferation, migration and differentiation of normal and IPF lung fibroblasts. Furthermore, both drugs reversed TGF-β1-induced effects on MMP-1, − 2, − 3, − 7, and − 9, while only PFD promoted TIMP-1 and-2 expression and release.

**Conclusions:**

Our findings demonstrate that the antifibrotic effects of IFN-γ and PFD on normal and IPF lung fibroblasts are different and complementary. Combination therapy with inhaled IFN-γ and PFD in IPF is promising and should be further explored in IPF clinical trials.

## Introduction

Idiopathic pulmonary fibrosis (IPF) is the most common form of idiopathic interstitial pneumonia, and patients with IPF exhibit poor survival and have limited therapeutic options [1, 2]. Dysregulated wound healing is thought to cause the development of fibrosis through excess fibroblast proliferation, migration, and differentiation to myofibroblasts and extracellular matrix (ECM) deposition [3]. In the IPF lung, aggregates of proliferating fibroblasts and myofibroblasts, termed “fibroblast foci”, are a key histopathological finding that indicates ongoing fibrosis [4]. Targeting the inhibition of key fibroblast activities may lead to novel therapeutic approaches for IPF [5–7]. ECM remodeling and degradation is maintained by the balance of matrix metalloproteinases (MMPs) and tissue inhibitors of matrix metalloproteinase (TIMPs) [8–13]. MMPs not only degrade all the components of the extracellular matrix, but they are also able to release, cleave and activate a wide range of growth factors, cytokines, chemokines and cell surface receptors affecting numerous fibroblasts functions, including adhesion, proliferation, differentiation, recruiting and transmigration, and apoptosis [11, 14–16].

Given the complex pathogenesis of IPF leading to lung fibrosis, which involves multiple coactivated pathways, combination therapy may be a rational treatment approach in IPF patients. Treatment of IPF with pirfenidone or nintedanib does not stop ongoing fibrosis and is limited to patients with only mild to moderate disease [17]. Pirfenidone (PFD) is a pleiotropic molecule that inhibits TGF − β, collagen synthesis, and fibroblast proliferation and mediates tissue repair [18–20]. The molecular mechanisms responsible for the antifibrotic action of PFD are still under study.

Interferon-γ (IFN-γ) is a pleiotropic cytokine with antiviral, antibacterial, antitumor, and pro-inflammatory properties that also has antifibrotic activities [21]. Because of the latter, IFN-γ is a potential effective biological agent to treat not only lung fibrosis but also liver and renal fibrosis [22–26]. Microarray analysis of the lung fibroblasts from fibrotic lungs showed the strong suppression of IFN-γ-stimulated genes [27]. We recently demonstrated that inhaled IFN-γ in IPF patients is safe with no systemic side effects, an effect very different from that of systemic IFN-γ, which causes generalized systemic side effects [21]. Furthermore, we found that inhaled IFN-γ was associated with a significant improvement in lung diffusion capacity [24, 28, 29].

The present study aims to examine the in vitro effect of individual and combined treatment with IFN-γ and PFD on the activation of NHLF and IPF fibroblasts and ECM remodeling after TGF-β1 and PDGF-BB stimulation.

Here, we show that IFN-γ treatment attenuates normal and IPF fibroblast differentiation to the myofibroblast phenotype in vitro more effectively than PFD, illuminating a potential therapeutic strategy. Furthermore*,* IFN-γ in combination with PFD has a possibly synergistic/additive effect by inhibiting the activation and differentiation of NHLF and IPF lung fibroblasts possibly by altering the balance of MMPs and TIMPs.

## Methods

### Cell culture

A normal human lung fibroblast (NHLF) cell line (cat # CC2512) was purchased from Lonza (Lonza, Walkersville, MD, USA). We used 3 different batches of NHLF cells collected from 3 different donors as follows: Batch # 0000343490 was collected from a 24-year-old female; batch # 0000369145 was collected from a 43-year-old male and batch # 0000608197 was collected from a 67-year-old male. NHLFs were cultured in fibroblast growth medium (FGM-2) (Lonza), and cells at passage 3 to 7 were used for all experiments. Three human IPF lung fibroblast cell lines (CCL-134 and CCL-191) were purchased from American Type Culture Collection (ATCC) (ATCC, Manassas, VA, USA), and IPF lung fibroblasts (cat # CC-7231) purchased from Lonza at passage 2 to 5 were used for all experiments. IPF lung fibroblasts from ATCC were cultured in F12K medium, while those from Lonza were cultured in FGM-2 (Lonza). Early passages of IPF fibroblasts were used in this study as IPF-derived fibroblasts are known to display morphological features of replicative senescence even at early passage, unlike age-matched control fibroblasts at the same passage [30, 31]. Human recombinant IFN-γ (Sigma-Aldrich, St. Louis, USA), PFD (TCI America, Montgomeryville, PA), TGF-β1 (Cell Signaling Technology, Danvers, USA) and PDGF-BB (R&D Systems, Minneapolis, USA) were used in our experiments.

### Treatment of IPF fibroblasts and NHLFs

Lung fibroblasts were seeded at a density of 20,000–25,000 cells/cm^2^, followed by starvation for 2 h in serum-free medium (SFM). The lung fibroblasts were subsequently treated with or without TGF-β1 (2.5 ng/ml) or PDGF-BB (20 ng/ml) with either IFN-γ (10–40 ng/ml), PFD (100–500 μg/ml) or both IFN-γ /PFD in serum-free media supplemented with 0.1% BSA to provide a protein source to support cell growth. We did not use serum in culture media because it has a mitogenic effect on fibroblasts. Previous reports showed that lung fibroblasts survived in serum-free medium and sustain an increase in cell number after 2 days of starvation in SFM secondary to the autocrine secretion of various mediators, including PDGF, that could influence proliferation and the synthetic activity of fibroblasts [32, 33]. IFN-γ was diluted to its final concentration in PBS. Cells received daily treatment with IFN-γ at the indicated doses because IFN-γ activity is stable for only 18 h in culture medium. PFD was dissolved in dimethyl sulfoxide (DMSO). The final DMSO concentration in the medium was always 1%.

### LDH cytotoxicity assay

Following treatment with different concentrations of PFD (100–500 μg/ml) and IFN-γ (10–40 ng/ml) at different time periods, the release of lactate dehydrogenase (LDH) was evaluated by the Colorimetric Cytotoxicity Detection Kit (BioVision Inc., Milpitas, CA, USA) according to the manufacturer’s instructions. Cytotoxicity (cell necrosis) was indicated as the percentage of LDH release calculated as the ratio between the mean absorbance of treated cultures minus the absorbance of untreated control cells and the mean absorbance of the highest value from the positive control (maximum LDH release by cell lysis) minus the absorbance of untreated cultures.

### Fibroblast proliferation studies

#### Cell titer 96® non-radioactive cell proliferation assay

NHLFs and IPF fibroblasts (5 × 10^4^) seeded on 24-well plates were grown overnight in serum-containing medium. On the following day, cells were switched to SFM supplemented with 0.1% BSA and treated with TGF-β1 (2.5 ng/ml) or PDGF-BB (20 ng/ml) and different concentrations of IFN-γ and PFD for 1, 3 or 5 days. After incubation, cell proliferation was assessed using the Cell Titer 96® Non-Radioactive Cell Proliferation Assay kit obtained from Promega Corporation (Promega Corp., Madison, WI, USA). It is basically a modified MTT assay that demonstrates less than a 5% difference in cell proliferation compared to [^3^H]-thymidine incorporation as per the Promega and our previous laboratory experience [34, 35]. The medium was replaced with fresh growth medium and dye solution, and cells were incubated at 37 °C for 2 h. After the addition of solubilization/stop solution, cells were incubated for another 1.5 h at 37 °C. The absorption of 100 μl aliquots in a Corning 96-well flat transparent plate at 570 nm was obtained using a plate reader.

#### Direct cell counts

NHLFs and IPF fibroblasts (5 × 10^4^) seeded on 24-well plates were grown overnight in serum-containing medium. On the following day, cells were switched to SFM supplemented with 0.1% BSA and treated with PDGF-BB (20 ng/ml) and different concentrations of IFN-γ and PFD for 1 or 3 days. At the end of the experiments, the cells were detached from the plate using BD cell detachment solution (Thermo-Fisher Scientific, Waltham, MA, USA). The cell number was counted by a Cellometer™ Auto 2000 cell counter (Nexcelom Bioscience, Lawrence, MA, USA). Cell viability was determined manually by counting cells after trypan blue staining using a Neubauer hematocytometer and an automated Cellometer.

### Migration assay using a modified Boyden chamber assay

NHLFs and IPF fibroblasts were assessed for their ability to migrate in the presence PDGF-BB (20 ng/ml) using a modified Boyden chamber assay. The migration assays were performed with 24-well tissue culture Transwell plates (Costar, Corning, NY, USA) composed of polycarbonate membranes with 8 μm pores. Lung fibroblasts were seeded on the upper chambers of the Transwells at 1 × 10^5^ cells in 100 μl of DMEM containing 0.1% BSA and either IFN-γ (10–40 ng/ml), PFD (100–500 μg/ml) or both IFN-γ /PFD. PDGF-BB (20 ng/ml) was added to the lower chambers. The Transwells were incubated for 24 h. at 37 °C in a CO_2_ incubator. The number of cells that migrated to the lower surface of the membrane was counted under 200× magnification. Ten random high-power fields were counted per sample. Each group was run in triplicate.

### Sircol assay

Confluent serum-deprived fibroblasts were stimulated with TGF-β1 (2.5 ng/ml) and incubated in the presence of either IFN-γ, PFD or both for the indicated time periods. Soluble collagen secretion (collagens type I to V) in the conditioned media was determined using a Sircol™ Assay kit (Biocolor; Carrick Fergus, UK) according to the manufacturer’s instructions.

### Western blotting

The protein concentrations of the cell lysates were determined by BCA assay. Equal amounts of proteins (20 μg) from cell lysates were resolved by 8–16% SDS-PAGE and transferred onto a nitrocellulose membrane (Amersham Biosciences, Pittsburgh, USA). After blocking with 5% milk, the membranes were incubated with primary antibodies overnight at 4 °C followed by incubation with horseradish peroxidase-conjugated secondary antibodies and detection by use of an enhanced chemiluminescence detection system (Amersham Bioscience, Pittsburgh, USA). Primary antibodies included mouse monoclonal anti-α-SMA (1:10000 dilution, Sigma-Aldrich, St. Louis, USA); rabbit polyclonal anti-MMP-1, MMP-3 and MMP-7 (1:1000 dilution, Sigma-Aldrich, St. Louis, USA); and β-tubulin (1:1000 dilution; Cell Signaling Technology, Danvers, USA). Bands were digitalized, and their densities were quantified using image analysis software. The results are expressed as a ratio of band density to total β-tubulin density.

### Gelatin zymography

Gelatin zymography of conditioned media was performed as described previously [34]. Briefly, conditioned media from the different treatment conditions were diluted 1:1 in nonreducing sample buffer and separated on 10% SDS polyacrylamide gels containing 0.1% gelatin (Thermo-Fisher Scientific, Waltham, MA USA) for 150 min at 125 V. SDS was removed by incubation with renaturing buffer (2.5% Triton X-100 diluted in water) for 30 min at room temperature. The gels were washed for 30 min in developing buffer (Thermo-Fisher Scientific, Waltham, MA USA) and then incubated overnight at 37 °C in fresh developing buffer. Finally, the gels were stained with Coomassie blue. Zones of enzymatic (gelatinolytic) activity were characterized by the absence of Coomassie blue staining.

### RNA extraction and qPCR

RNAs were extracted from IPF and normal lung fibroblasts 24 h and 72 h after TGF-β1 treatment with either IFN-γ, PFD or both using RNeasy mini kits (Qiagen; Valencia, CA, USA) according to the manufacturer’s instructions. The RNAs were reverse transcribed into cDNAs, which were subjected to qPCR analyses using primer pairs specific to each of the following genes*: COL1A1, ACTA2, MMP1, MMP3, MMP7, MMP8, MMP9, TIMP1, TIMP2* and *YWHAZ* (TaqMan™ Gene Expression Assay (FAM), Thermo-Fisher Scientific, Waltham, MA USA). qPCR was performed on an ABI Prism 7000 sequence detection system, and mRNA levels for each gene were analyzed with ABI Prism 7000 software (Applied Biosystems, Waltham, MA USA). Relative quantification of each target gene was performed using the comparative CT (2^-∆∆CT^) method with *YWHAZ* (*tyrosine 3 monooxygenase/tryptophan 5-monooxygenase activation protein, zeta polypeptide) used as a housekeeping gene for the* normalization of mRNA expression levels as reported in previous studies [36, 37]. Data were plotted as 2^-ΔΔCT^ (mean fold change) using Graph Pad prism software.

### Statistical analysis

All results are reported as the mean ± SEM. Data from image analysis are shown as representative immunoblots and as the means ± SEMs after densitometric image analysis. A paired t-test was used for statistical analysis between 2 groups, and ordinary one-way ANOVA followed by Tukey’s multiple comparisons test using GraphPad Prism 8 software were used for comparisons between groups to assess differences among conditions. Significance was indicated when *p < 0.05.

## Results

### IFN-γ and PFD attenuate the proliferation of NHLFs and IPF fibroblasts in response to TGF-β1 and PDGF-BB

**Fig. 1:**
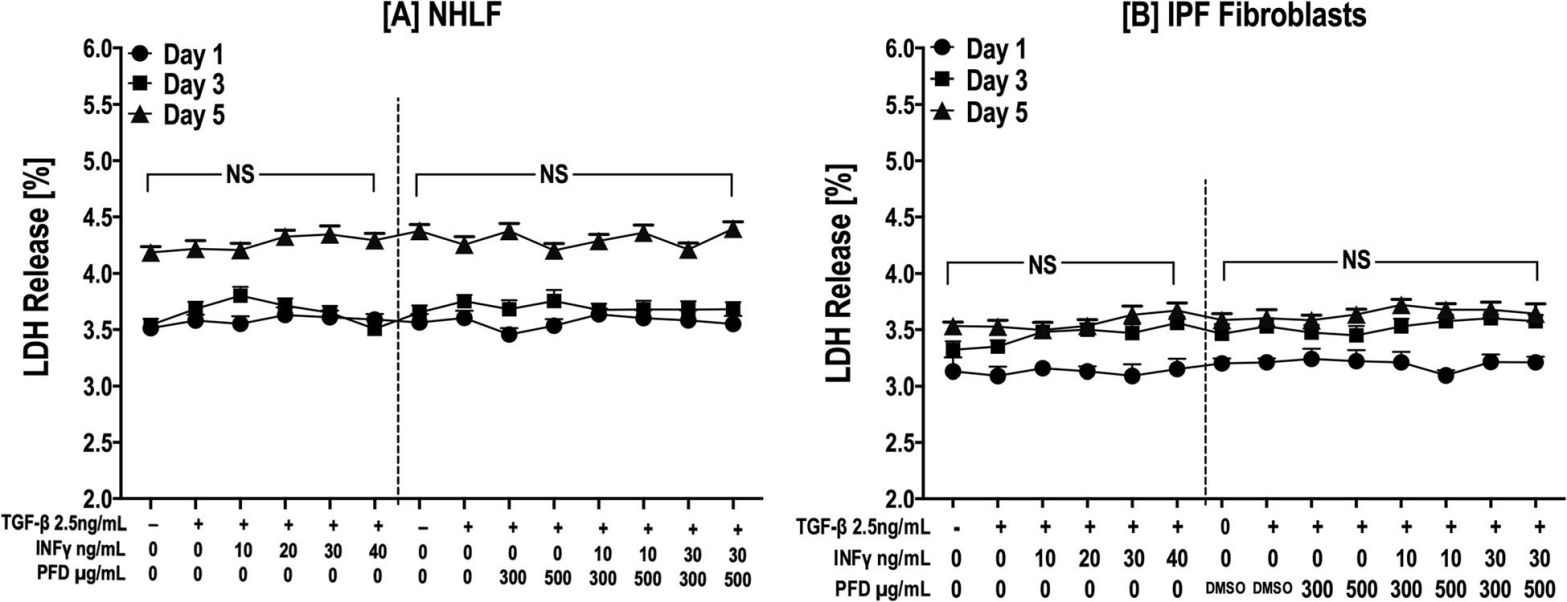
Effect of IFN-γ, PFD and their combination on the viability of NHLFs and IPF fibroblasts. NHLFs (**a**) and IPF fibroblasts (**b**) were incubated with INF-γ [10–40 ng/mL], PFD [300–*500* μg/ml] and *a* combination of NF-γ /PFD in the presence of TGF − β1 [2.5 ng/mL] for 1, 3 and 5 days. Cell viability was determined by LDH release cytotoxicity assay. Data are presented as *the mean* ± SEM of the % LDH released to that of control samples in three different experiments, with each experiment run in duplicate. [NS] comparing treatment to control media or control media with vehicle [DMSO]

For in vitro experiments, we selected a range of IFN-γ (10–40 ng/mL) and PFD (100–500 μg/mL) concentrations similar to those used in published studies [38–42]. NHLFs and IPF fibroblasts derived from different cell lines and from different batches were used in all experiments. NHLFs and IPF fibroblasts were incubated with INF-γ, PFD and a combination of IFN-γ/PFD in the presence of TGF − β1 (2.5 ng/mL) or PDGF-BB (20 ng/ml) for 1, 3 and 5 days. Initially, cell viability was measured with an LDH release cytotoxicity assay, Trypan blue staining and automated cell counting using a Cellometer Auto 2000 in some experiments. IFN-γ, PFD or both at the concentrations tested had no cytotoxic effects on NHLFs and IPF fibroblasts for a treatment period of 1, 3 and 5 days (Fig. 1a & b).

**Fig. 2:**
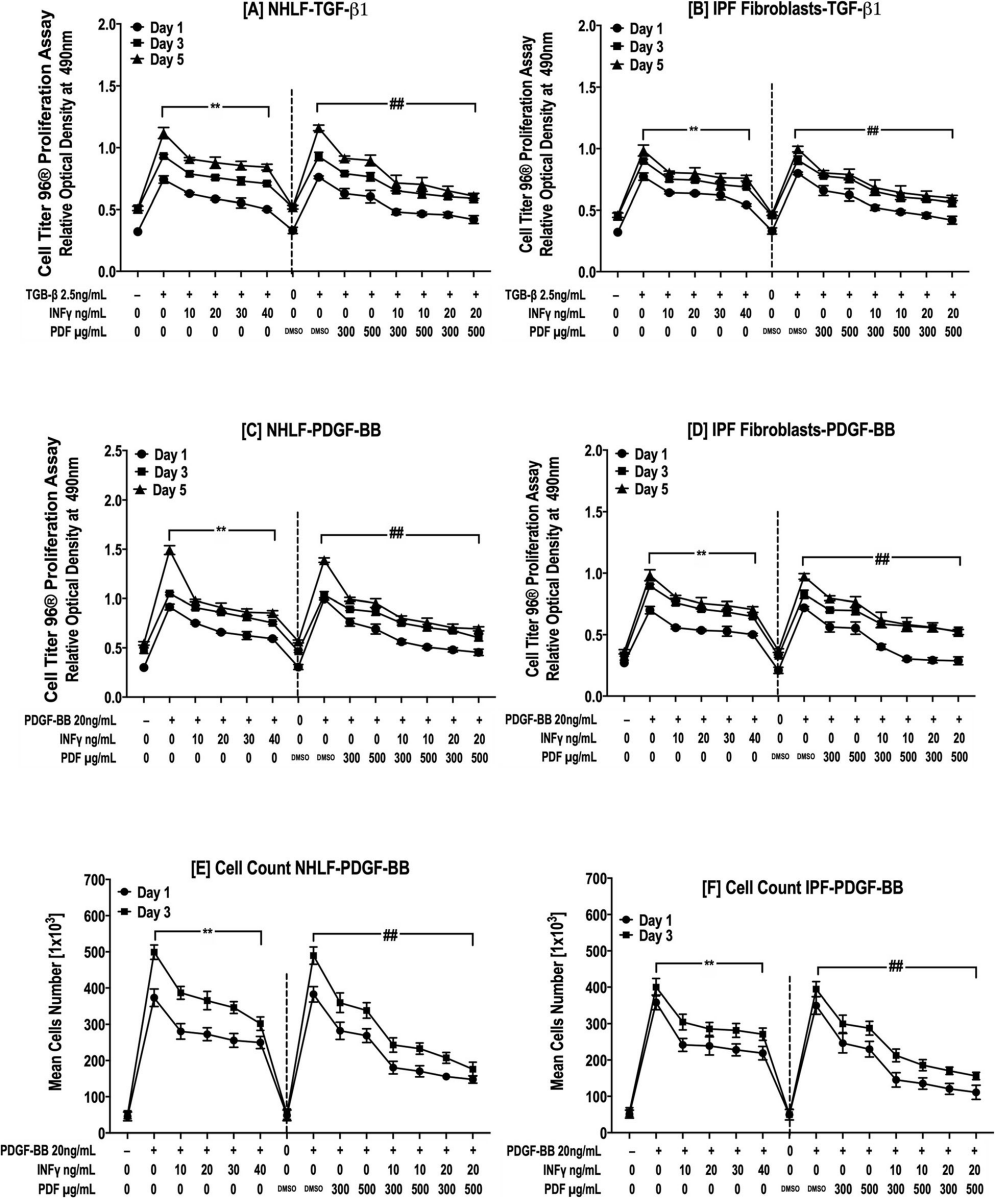
INF-γ and PFD inhibit the proliferation of NHLFs and IPF fibroblasts in response to TGF-β1 and PDGF-BB. NHLFs and IPF fibroblasts were stimulated with either TGF-β1 2.*5 ng*/ml (**a** & **b**) or PDGF-BB 20 ng/ml (**c-f**) in the presence of INF-γ [10–40 ng/mL], PFD [300–*500* μg/mL] or *a* combination of IFN-γ/PFD for 1, 3 and 5 days. Cell Titer 96® Non-Radioactive Cell Proliferation Assay (**a-d**) and cell counting using trypan blue staining and an automated Cellometer (**e** & **f**) revealed the proliferative response to both TGF-β1 and PDGF-BB when compared with proliferation of the control, and both IFN-γ and PFD alone inhibited TGF-β1*-* and PDGF-BB*-*induced proliferation in both NHLFs and IPF *fibroblasts. The combination* of both IFN-γ and PFD treatment restrained cell proliferation to control levels. Data are presented as the means ± SEMs of 3 independent experiments, with each experiment run in duplicate. ^**^P < 0.05 vs. TGF-β1 and ^##^ *P* < 0.05 vs. TGF-β1+ vehicle [DMSO]

Next, NHLF and IPF fibroblast cell proliferation was examined using a Cell Titer 96® Non-Radioactive Proliferation Assay (Promega Corp., Madison, USA) or automated cell counting. As shown in Fig. 2a & b, TGF-β1-induced proliferation of NHLFs and IPF lung fibroblasts was significantly reduced by IFN-γ (10–40 ng/ml) and PFD (300–500 μg/ml) in a dose-dependent manner after 1, 3 and 5 days (*p* = 0.0002, Fig. 2a & *p* = 0.014 Fig. 2b). While no significant difference in cell proliferation was noted between IFN-γ and PFD treatment, the combination of both drugs significantly inhibited the TGF-β1-induced proliferation of NHLFs and IPF fibroblasts compared to individual drugs (Fig. 2a & b).

When 20 mg/ml PDGF-BB was used as a mitogen, as shown in Fig. 2c-f, both IFN-γ and PFD significantly inhibited the PDGF-BB-induced proliferation of NHLFs and IPF fibroblasts. Furthermore, a significant difference in the effect of individual and combined treatment with both IFN-γ and PFD was observed (*p* < 0.01, Fig. 2c-f). Interestingly, combination therapy with both IFN-γ and PFD, especially at high doses, attenuated cell proliferation to control levels (*p* > 0.05, Fig. 2a-f). The mitogenic effect of PDGF-BB was noticeably more potent in NHLFs compared to that in IPF fibroblasts, which is consistent with previous data demonstrating the different responses of normal and IPF fibroblasts to PDGF-BB and other growth factors [33, 43]. The enhanced mitogenic effect of PDGF-BB on normal compared to fibrotic fibroblasts in lung fibrosis models was attributed to the upregulated expression of PDGFRα [33].

### IFN-γ and PFD inhibit NHLF and IPF fibroblast migration in response to PDGF-BB with combined therapy exhibiting a synergistic effect

**Fig. 3:**
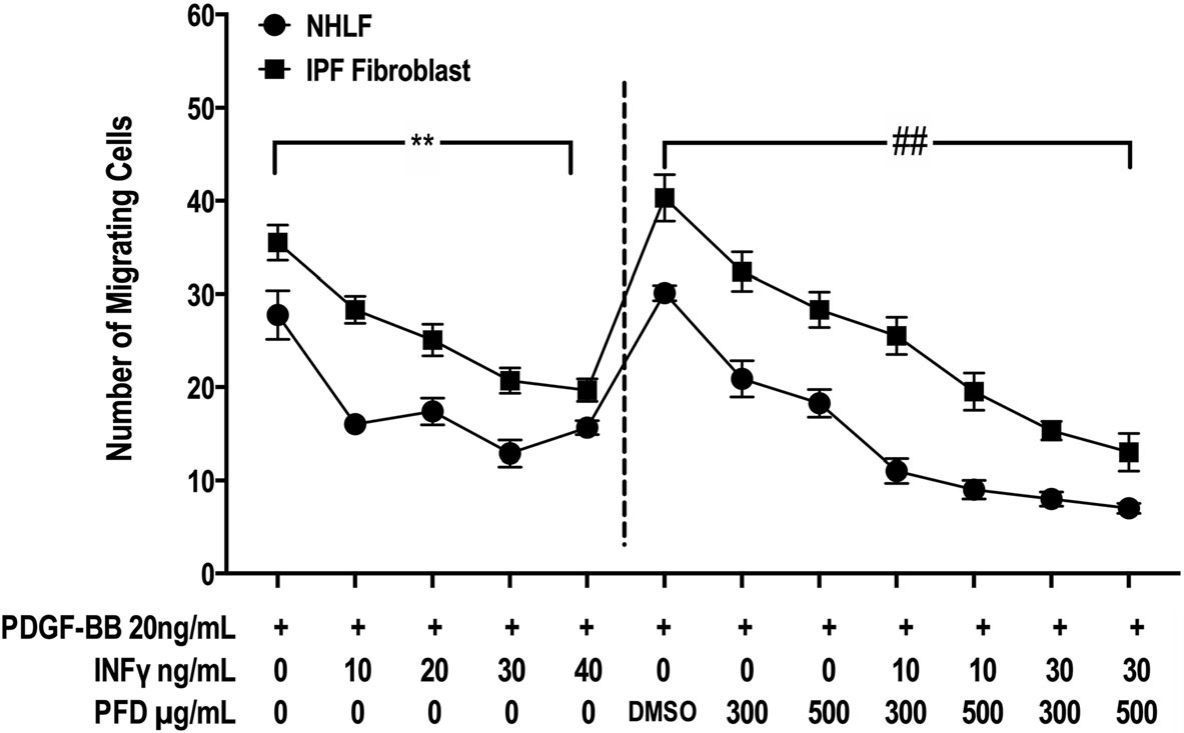
INF-γ and PFD inhibit the migration of NHLFs and IPF fibroblasts. Cell migration was assessed using *a modified* Boyden chamber in NHLFs and IPF fibroblasts treated with either INF-γ [10–40 ng/ml], PFD [300–500 μg/ml] or *a* combination of IFN-γ/PFD in *the* upper chamber and PDGF-BB *20 ng*/ml used as a chemoattractant in *the* lower chamber for 24 h. Data are presented as the means ± SEMs of 3 independent experiments each run in *duplicate*. ^**^P < 0.05 vs. control PDGF-BB and ^##^ *P* < 0.05 vs. PDGF-BB + vehicle [DMSO]

Both IFN-γ (10–40 ng/ml) and PFD (300–500 μg/ml) inhibited the migration of NHLFs and IPF fibroblasts in a dose-dependent manner in the presence of PDGF-BB (20 ng/ml) (Fig. 3). IFN-γ (20–40 ng/ml) significantly inhibited normal and IPF fibroblast migration compared to PFD (300–500 μg/ml) treatments. Furthermore, the combination of both IFN-γ and PFD significantly inhibited fibroblast migration in response to PDGF-BB compared to that following individual treatment, indicating the possibly synergistic effect of combination treatment (*p* < 0.001, Fig. 3).

### IFN-γ and PFD synergistically downregulate the differentiation of normal and IPF fibroblasts into myofibroblasts

**Fig. 4:**
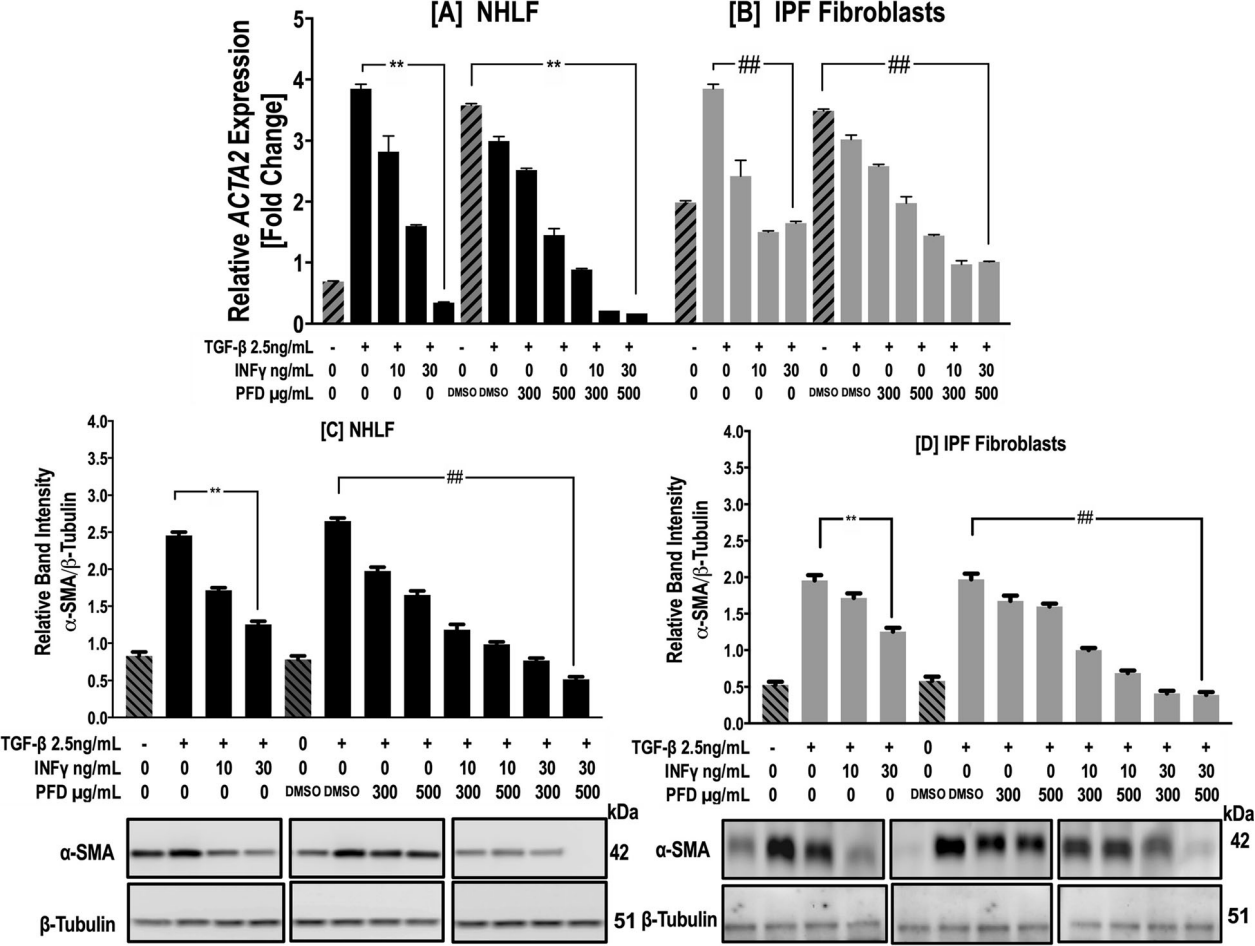
IFN-γ and PFD synergistically inhibit TGF-β1*-*induced differentiation of NHLFs and IPF fibroblasts into myofibroblasts. Levels of α-SMA*,* a marker of fibroblast to myofibroblast differentiation*,* were determined in NHLFs and IPF fibroblasts treated with INF-γ [10–30 ng/ml], PFD [300–*500 μg*/ml] or *a* combination of IFN-γ/PFD in the presence of TGF-β1 [2.5 ng/mL] for 3 days. Upper panels show *ACTA2* gene expression measured by qPCR from NHLFs (**a**) and IPF fibroblasts (**b**). Data for the relative quantification of each target gene are plotted as 2^-ΔΔCT^ (mean fold change) using *the YWHAZ housekeeping gene as a control.* Lower panels show densitometric analysis and representative blots of cell lysates from NHLFs (**c**) and IPF fibroblasts (**d**) for α-SMA with β-tubulin as a loading control. Data are presented as the mean ± SEM of 3 independent experiments, with each experiment run in duplicate. ^**^P < 0.05 vs. TGF-β1 and ^##^ *P* < 0.05 vs. TGF-β1+ vehicle [DMSO]

α-SMA expression, a hallmark of fibroblast differentiation to myofibroblasts, was examined using immunoblotting and qPCR. Both IFN-γ and PFD inhibited the TGF-β1-induced expression of *ACTA2* and α-SMA protein in both NHLFs and IPF fibroblasts (Fig. 4a-d). The effect of IFN-γ (30–40 ng/ml) was more potent than that of PFD (300–500 μg/ml) in attenuating normal and IPF fibroblast differentiation (*p* < 0.001, Fig. 4a-d). Compared to individual drug treatment, the combination of IFN-γ and PFD significantly inhibited TGF-β1-induced *ACTA2* and α-SMA expression in NHLFs and IPF fibroblasts (*p* < 0.01, Fig. 4a-d).

**Fig. 5:**
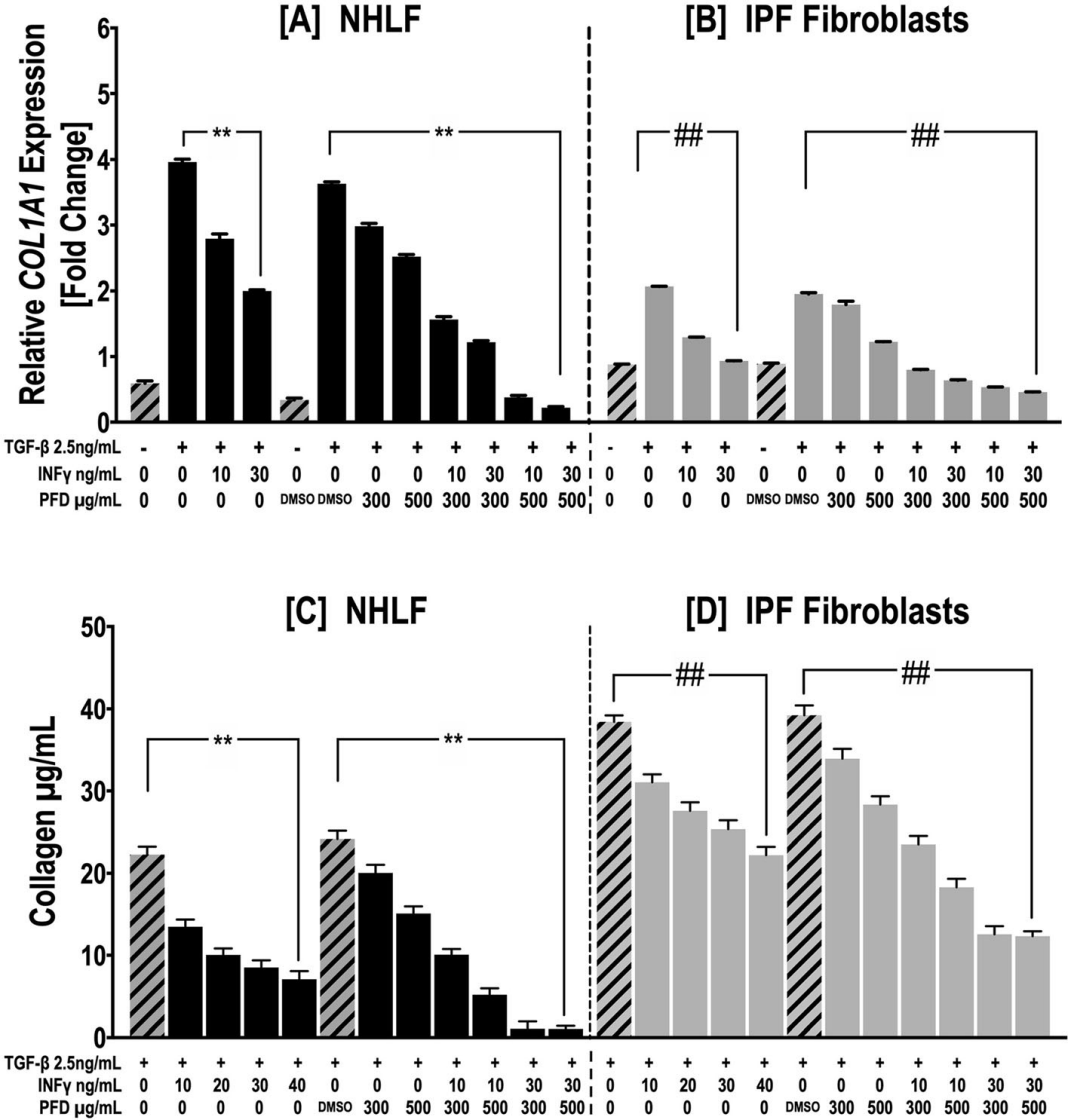
IFN-γ and PFD synergistically downregulate TGF − β1*-*induced collagen expression and release. NHLFs and IPF fibroblasts were treated with INF-γ [10–40 ng/mL], PFD [300–*500 μg*/ml] or *a* combination of IFN-γ/PFD in the presence of TGF − β1 [2.5 ng/ml] for 3 days. The upper panel shows *COL1A1* gene expression measured by quantitative RT-PCR from NHLFs (**a**) and IPF fibroblasts (**b**). Data for the relative quantification of each target gene are plotted as 2^-ΔΔCT^ (mean fold change) using *the YWHAZ housekeeping gene as a control.* The lower panel shows the collagen concentration in the conditioned media from NHLFs (**c**) and IPF fibroblasts (**d**) cultured for 3 days *as* measured by Sircol assay. Data are presented as the mean ± SEM of 3 independent experiments, with each experiment run in duplicate. ^*^P < 0.05 vs. TGF-β1 and ^#^ *P* < 0.05 vs. TGF-β1+ vehicle [DMSO], respectively

Aberrant deposition of type I collagen-rich ECM is the typical phenotype of activated fibroblast/myofibroblasts in IPF lesions. Therefore, *COL1A1* expression and the total soluble collagen content were examined using qPCR and a Sircol assay, respectively. Individual and combined IFN-γ and PFD treatment inhibited TGF-β1-induced *COL1A1* expression in both NHLFs and IPF fibroblasts, with a significant difference noted between the effects of IFN-γ and PFD treatment (*p* < 0.01, Fig. 5a & b) and individual and combined treatment (*p* < 0.001, Fig. 5a & b). Similar to *COL1A1* expression, secreted soluble collagen expression was consistently decreased by individual and combined IFN-γ and PFD treatment, with a significant difference observed between IFN-γ and PFD treatment and individual and combined treatment (Fig. 5c & d).

### IFN-γ and PFD reverse the effect of TGF-β1 on MMP/TIMP expression and release

**Fig. 6:**
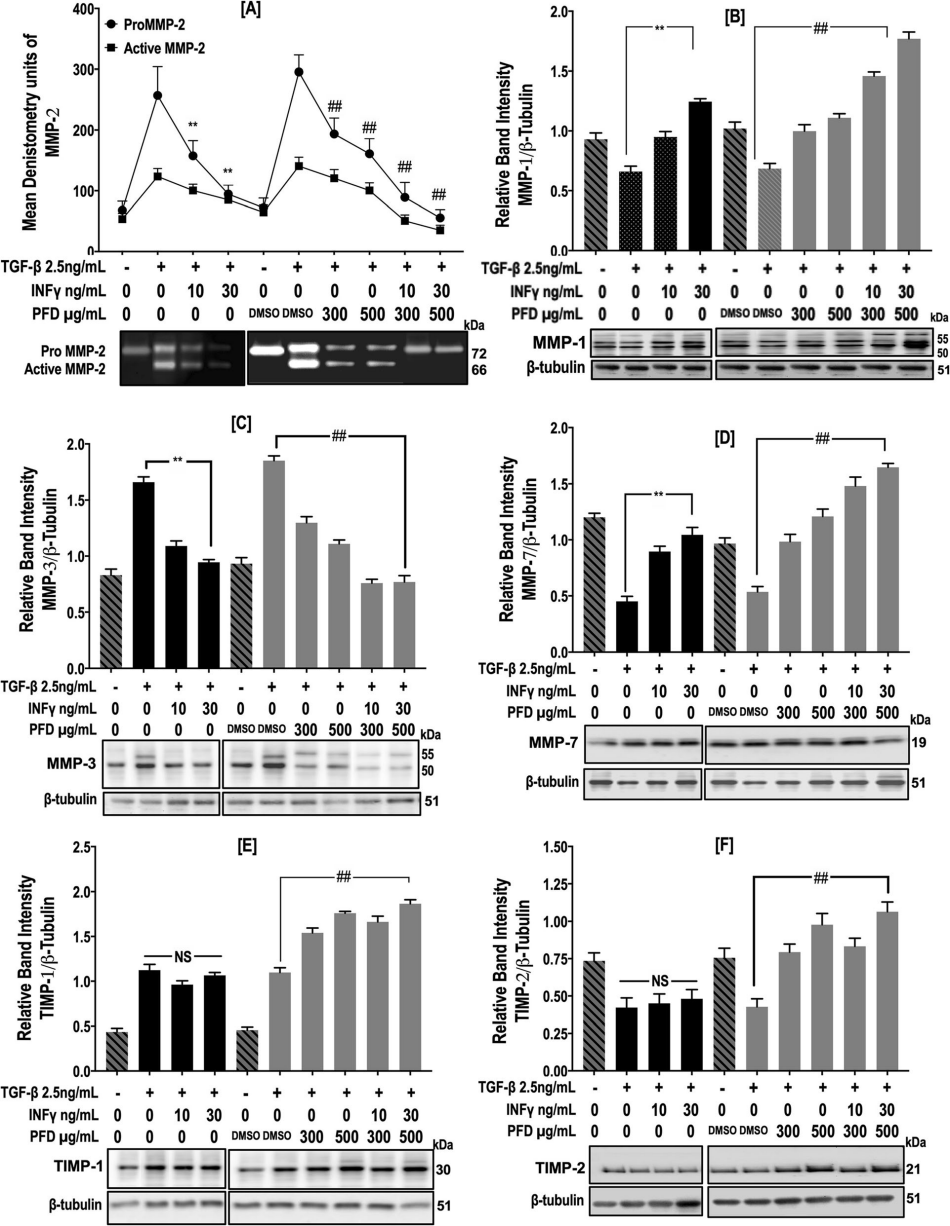
IFN-γ and PFD reverse the effects of TGF-β1 on MMP and TIMP release. NHLFs were treated with INF-**γ** [10–30 ng/ml], PFD [300–*500 μg*/ml] or *a* combination of IFN-γ/PFD in the presence of TGF − β1 [2.5 ng/ml] for 3 days. Conditioned media was examined for MMP-2 using gelatin zymography*,* while MMP-1, MMP-3, MMP-7, TIMP-1 and TIMP-2 were examined using Western blotting with β*-tubulin* as a loading control. Data following densitometric analysis with a representative zymogram of MMP-2 (**a**) and blots for MMP-1 (**b**), MMP-3 (**c**), MMP-7 (**d**), TIMP-1 (**e**) and TIMP-2 (**f**) are shown. Data are presented as the mean ± SEM of 3 independent experiments, with each experiment run in duplicate. ^**^P < 0.05 vs. TGF-β1 and ^##^ *P* < 0.05 vs. TGF-β1+ vehicle [DMSO]

MMP and TIMP expression and release were examined using qPCR, immunoblot analysis and gelatin zymography. TGF-β1 induced an increase in the intensity of the bands corresponding to proMMP-2 and active MMP-2. IFN-γ and PFD individually and in combination significantly inhibited TGF-β1-induced MMP-2 activation, as shown by gelatin zymography (Fig. 6a). Immunoblot analysis of supernatants with antibodies against MMP-1, MMP-3 and MMP-7 revealed that culture in the presence of TGF-β1 increased the intensity of the band corresponding to MMP-3, while the MMP-1 and -7 bands were decreased by TGF-β1 treatment. Individual and combined treatment with IFN-γ and PFD induced a concentration-dependent decrease in the abundance of MMP-3 and increase in MMP-1 and MMP-7 in supernatants (Fig. 6b-d). Immunoblot analysis with antibodies against TIMP-1 and -2 showed that TGF-β1 increased the intensity of the band for TIMP-1 and decreased that of the band for TIMP-2, whereas PFD either alone or in combination with IFN-γ induced a significant increase in the amounts of TIMP-1 and -2 (Fig. 6e & f). IFN-γ treatment had no significant effect on the intensity of the bands for TIMP-1 and -2 when compared to TGF-β1 treatment (Fig. 6e & f).

**Fig. 7:**
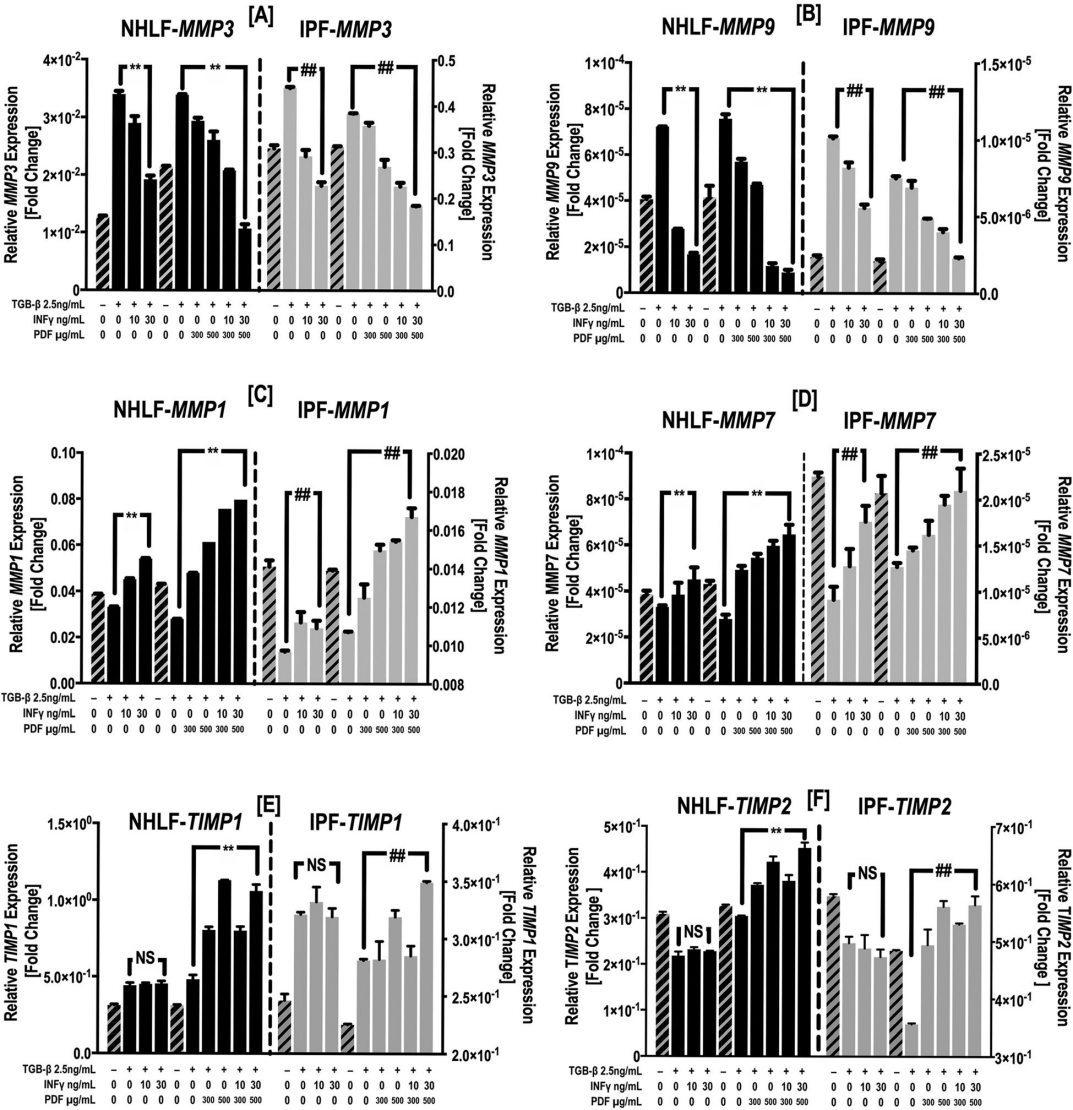
IFN-γ and PFD reverse the effect of TGF-β1 on MMP and TIMP gene expression. NHLFs [black bars, left y-axis] and IPF fibroblasts [gray bars, right y-axis] were treated with INF-γ [10–30 ng/ml], PFD [300–*500 μg*/ml] or *a* combination of IFN-γ/PFD in the presence of TGF − β1 [2.5 ng/ml] for 3 days. *MMP3* (**a**), *MMP9* (**b**)*, MMP1* (**c**)*, MMP7* (**d**)*, TIMP1* (**e**) and *TIMP2* (**f**) gene *expression was* measured by qPCR. Data for the relative quantification of each target gene are plotted as 2^-ΔΔCT^ (mean fold change) using *the YWHAZ housekeeping gene as a control.* Data are presented as the mean ± SEM of 3 independent experiments, with each experiment run in duplicate. ^**^P < 0.05 vs. TGF-β1 and ^##^ *P* < 0.05 vs. TGF-β1+ vehicle [DMSO]

Real-time PCR also showed that individual and combined treatment *with* IFN-γ and PFD reversed the effects of TGF-β1 on *MMP* expression and resulted in a dose-dependent decrease in *MMP3* and *MMP9* (Fig. 7a & b). In contrast, *MMP1* and *MMP7* mRNA expression was increased by IFN-γ and PFD as well as the combination of both (Fig. 7c-d). Furthermore, TGF-β1 treatment upregulated *TIMP1* mRNA levels and downregulated *TIMP2* mRNA levels*,* and only PFD treatment*,* either alone or in combination with IFN-γ, resulted in an increase in both *TIMP1* and *TIMP2* mRNA levels. IFN-γ treatment had no significant effect on the TGF-β1-induced regulation of *TIMP1* and *TIMP2* expression (Fig. 7e & f).

## Discussion

PFD and nintedanib are the only approved treatments for IPF. Both drugs have limited therapeutic value because they only slow *the* progression of fibrosis and produce systemic side effects. Although previous clinical trials of the systemic administration of IFN-γ in IPF patients failed [21, 44, 45]*, our* group has demonstrated that inhaled IFN-γ is safe and without systemic effects, and inhaled IFN-γ produced a measured improvement in the diffusion capacity in patients with IPF [28, 29]. In this context, this study was designed to examine *the* in vitro antifibrotic effect of combined IFN-γ and PFD treatment on NHLFs and IPF lung *fibroblasts* stimulated with either TGF-β1 or PDGF-BB.

The present study demonstrates that both IFN-γ and PFD attenuate the activation of NHLFs and IPF fibroblasts at several regulatory levels, including the inhibition of lung fibroblast proliferation, migration, differentiation to myofibroblasts and collagen secretion, after TGF-β1 and PDGF-BB stimulation. Compared to PFD, the effect of IFN-γ in downregulating lung fibroblast differentiation to *myofibroblasts is clearly more potent,* as evidenced by a reduction in the expression *of* α-SMA *and* collagen 1 and the attenuation of total collagen secretion. More importantly, the combination of both drugs had a possibly synergistic inhibitory effect on lung fibroblast proliferation, migration and differentiation. *Furthermore*, both IFN-γ and PFD *reversed* the effect of TGF-β1 on MMP-1, − 2, − 3, − 7 and − 9*,* while only PFD *increased TIMP1* and *TIMP2* expression and release. Collectively, these findings indicate that IFN-γ has antifibrotic properties in vitro and *that* its combination with PFD has a possibly synergistic antifibrotic effect on NHLFs and IPF *fibroblasts* by altering the balance of MMPs and TIMPs.

Both IFN-γ and PFD inhibited the TGF-β1*-* and PDGF-BB*-*induced proliferation of normal and IPF lung fibroblasts. Consistently, previous reports have shown that PFD and IFN-γ modulate TGF-β1*-*induced proliferation in not only normal lung fibroblasts [46–48] but also fibroblasts isolated from IPF patients [49, 50]. Moreover, the present study demonstrates that the combination of IFN-γ and PFD has a synergistic attenuating effect on the TGF-β1*-* and PDGF-BB*-*induced proliferation of normal and IPF fibroblasts (Fig*.* 2a-f).

Here*,* we show that the INF-γ-induced downregulation of TGF-β1-induced fibroblast differentiation to myofibroblasts was more potent than that induced by PFD (Fig. 4a-d). Previous reports showed that INF-γ has *a* strong inhibitory effect on the expression of α-SMA in both in vivo [41, 51] and in vitro models of fibrosis*.* These findings suggested that INF-γ exerts its antifibrotic effects in part via the inhibition of fibroblast differentiation to myofibroblasts [52, 53]. By inhibiting fibroblast differentiation to myofibroblasts, IFN-γ treatment results in the inhibition of *COL1A1* gene expression and total collagen secretion (Fig. 5a-d) in a manner similar to that suggested by previously reported data [50, 54, 55]. Importantly, we found that combined IFN-γ and PFD significantly inhibited fibroblast differentiation and collagen expression and release. This finding suggests that both IFN-γ and PFD carry out their antifibrotic actions via different mechanisms of action. PFD has been reported to inhibit fibroblast differentiation [18, 39, 46, 47, 49, 56] through dual hedgehog (Hh) pathway/TGF-β1 inhibition by targeting the GLI2 protein [19]. In contrast, IFN-γ inhibits fibroblast differentiation to myofibroblasts by antagonizing TGF-β1 signaling in part via the upregulation of SMAD7 [42, 53] and the antagonistic interaction of SMAD and the JAK pathway at the nuclear p300/CBP level [57, 58].

Matrix metalloproteinases (MMPs) and their inhibitors (TIMPs) have been implicated in the pathogenesis of IPF. TGF-β1 is known to *regulate* the expression of several MMPs in lung fibroblasts, which in turn can participate in TGF-β1 activation [15, 59–61]. This study presents evidence that both IFN-γ and PFD exert antifibrotic effects in part by reversing the effect of TGF-β1 on MMPs and altering the balance of MMPs and TIMPs. Both IFN-γ and PFD inhibit the TGF-β1*-*induced activation of MMP-2 (Fig. 6a) and *MMP9* gene expression (Fig. 7b). MMP-2 and -9 are known to activate latent ECM-bound TGF-β1, contributing to an enhanced pool of active TGF-β1 [15, 60]. MMP-9 is expressed by lung fibroblasts in fibroblastic foci [11] induced mainly by TGF-β1 as part of a fibrogenic feedback loop in IPF lungs [62]. MMP-9 exerts its fibrotic effect by cleaving insulin-like growth factor (IGF)–binding protein-3 (IGFBP-3). IGFBP-3 serves as a downstream modulator of TGF-β1 by inducing the production of syndecan-2 of fibroblasts [63, 64]. Syndecan-2 promotes cell signaling, proliferation, migration, cytoskeletal organization, cell-matrix interactions, and ECM assembly in lung fibroblasts [65]. Furthermore, both IFN-γ and PFD inhibit TGF-β1-induced *MMP3* upregulation (Figs. 6c and 7a). MMP-3 expressed by lung fibroblasts plays a role in promoting EMT in lung epithelial cells via activating β-catenin signaling [12] and activating latent TGF-β1 [66]. In support of our data, previous studies demonstrated the inhibitory effect of PFD on the expression of *MMP3* [20], *MMP2* and *MMP9* [67, 68]. Other reports also demonstrated that IFN-γ significantly attenuates IL-1-induced *MMP1* and *MMP3* production but has no effect on TIMP-1 production in rheumatoid arthritis*-*derived fibroblasts [40].

Both IFN-γ and PFD reversed the TGF-β1*-*induced inhibition of *MMP7* and *MMP1* expression. MMP-1 is significantly overexpressed in IPF lungs [11] and was shown to have in vitro protective activities *against* IPF fibroblasts [14]. Moreover, MMP-1 in lung epithelial cells increases their proliferation, migration, and expression of hypoxia-inducible factor-1α [8, 69]. In contrast, MMP-7 carries out both pro- and antifibrotic functions in lung fibrosis. Early on, MMP-7 functions by facilitating neutrophil influx and activation, leading to epithelial damage and an enhanced fibrotic environment. Then, epithelial-derived MMP-7 promotes resolution by attracting an influx of immunosuppressive leukocytes [70].

*Interestingly*, only PFD treatment increased *TIMP1* and *TIMP2* expression and release in normal and IPF fibroblasts *(*Figs. 6 and 7). TIMP-*1 and* -2 are highly induced during fibrosis in a number of animal models of lung fibrosis [71–73]. In fact, studies using mice genetically deficient for different TIMPs have demonstrated that TIMPs play many different roles, one of which is restricting ECM deposition and reducing ECM abundance by controlling cell function [74–77]. Although *TIMP1*^*−/−*^ *mice* have pulmonary fibrotic responses to bleomycin similar to those of wild*-*type mice [78], inflammation is significantly increased in *TIMP1*^*−/−*^ mice*,* suggesting that TIMP-1 plays a key role in restricting inflammation following lung injury [78]. TIMP-1 plays a similar role in the liver, as *TIMP1*^*−/−*^ mice had significantly increased injury, inflammation, and fibrosis following carbon tetrachloride-induced liver injury compared to wild*-*type mice [79].

Taken together, the data presented here *provide* evidence that IFN-γ and PFD have different and potentially complementary antifibrotic effects on NHLFs and IPF lung fibroblasts. IFN-γ exerts its antifibrotic effect by inhibiting fibroblast differentiation to myofibroblasts and ultimately inhibits collagen and α-SMA expression possibly via counteracting the effects of TGF-β1. In contrast, PFD exerts its antifibrotic effect on lung *fibroblasts* possibly by reversing *the effect of* TGF-β1 on MMPs/TIMPs. IFN-γ and PFD in combination have a possibly synergistic effect on lung fibroblast activation and *differentiation* to myofibroblasts. Unfortunately, this in vitro study is still far from able to simulate complex situations such as the interactions between cell types and their compensatory mechanisms. The present findings support further investigation of the mechanisms underlying *the* antifibrotic effect of combined IFN-γ and PFD therapy *in an* in vivo model of lung fibrosis.

## Conclusion

The present study demonstrates that combined IFN-γ and PFD treatment has possibly synergistic antifibrotic effects on NHLFs and IPF *fibroblasts.* Combination therapy with IFN-γ and PFD may be a new and promising approach to the treatment of IPF and should be further explored in clinical trials.

## Data Availability

The datasets used and/or analyzed during the current study are available from the corresponding author on reasonable request.

## Abbreviations

*ACTA2*: α-Smooth Muscle Actin
*COL1A1*: Collagen-1α1
ECM: Extracellular Matrix
EMT: Epithelial Mesenchymal Transformation
IFN-γ: Interferon-γ
IGFBP-3: Insulin Growth Factor Binding Protein-3
IPF: Idiopathic Pulmonary Fibrosis
MMP: Matrix Metalloproteinase
NHLF: Normal Human Lung Fibroblast
PDGF-BB: Platelet Derived Growth Factor-BB
PFD: Pirfenidone
qPCR: Quantitative Polymerase Chain Reaction
TGF-β1: Transforming Growth Factor-β1
TIMP: Tissue Inhibitors of Matrix Metalloproteinase
α-SMA: α-Smooth Muscle Actin
